# SNV discovery and functional candidate gene identification for milk composition based on whole genome resequencing of Holstein bulls with extremely high and low breeding values

**DOI:** 10.1371/journal.pone.0220629

**Published:** 2019-08-01

**Authors:** Shan Lin, Hongyan Zhang, Yali Hou, Lin Liu, Wenhui Li, Jianping Jiang, Bo Han, Shengli Zhang, Dongxiao Sun

**Affiliations:** 1 Department of Animal Genetics and Breeding, College of Animal Science and Technology, Key Laboratory of Animal Genetics and Breeding of the Ministry of Agriculture, National Engineering Laboratory for Animal Breeding, China Agricultural University, Beijing, China; 2 Laboratory of Disease Genomics and Individualized Medicine, Beijing Institute of Genomics, Chinese Academy of Sciences, Beijing, China; 3 Beijing Dairy Cattle Center, Beijing, China; HudsonAlpha Institute for Biotechnology, UNITED STATES

## Abstract

We have sequenced the whole genomes of eight proven Holstein bulls from the four half-sib or full-sib families with extremely high and low estimated breeding values (EBV) for milk protein percentage (PP) and fat percentage (FP) using Illumina re-sequencing technology. Consequently, 2.3 billion raw reads were obtained with an average effective depth of 8.1×. After single nucleotide variant (SNV) calling, total 10,961,243 SNVs were identified, and 57,451 of them showed opposite fixed sites between the bulls with high and low EBVs within each family (called as common differential SNVs). Next, we annotated the common differential SNVs based on the bovine reference genome, and observed that 45,188 SNVs (78.70%) were located in the intergenic region of genes and merely 11,871 SNVs (20.67%) located within the protein-coding genes. Of them, 13,099 common differential SNVs that were within or close to protein-coding genes with less than 5 kb were chosen for identification of candidate genes for milk compositions in dairy cattle. By integrated analysis of the 2,657 genes with the GO terms and pathways related to protein and fat metabolism, and the known quantitative trait loci (QTLs) for milk protein and fat traits, we identified 17 promising candidate genes: *ALG14*, *ATP2C1*, *PLD1*, *C3H1orf85*, SNX7, *MTHFD2L*, *CDKN2D*, *COL5A3*, *FDX1L*, *PIN1*, *FIG4*, *EXOC7*, *LASP1*, *PGS1*, *SAO*, *GPLD1* and *MGEA5*. Our findings provided an important foundation for further study and a prompt for molecular breeding of dairy cattle.

## Introduction

Milk yield, milk protein and fat traits are main economic traits and important breeding goals of dairy industry. Compared to the standard phenotypic data based methods, marker-assisted selection is expected to lead faster genetic progress by using information at the DNA level. Of note, genomic selection (GS) with the application of high density SNP chips has become the most popular and efficient technology in dairy cattle breeding since the first report of GS in 2001 by Meuwissen et al [1, 2]. Using publicly available quantitative trait loci (QTLs) and genome-wide association study (GWAS) data can improve the accuracy of whole genome prediction (WGP) compared to the chip-based GBLUP and BayesB methods et al[3]. Over the last few decades, with linkage (LA) or linkage and linkage disequilibrium (LA/LD) analysis, candidate genes approach and genome-wide association analysis (GWAS)[4], a great amount of QTLs and genetic associations for milk yield and milk composition have been identified in dairy cattle since the first report of QTL mapping in Holstein by Georges et al [5]. So far, the Cattle QTL database contains 3,996, 17,677, and 19,895 loci for milk yield, milk protein and fat, respectively (December 23, 2018, http://www.animalgenome.org/cgi-bin/QTLdb/). Nonetheless, merely *DGAT1*, *GHR*, and *ABCG2* gene have been validated to be true major genes for milk composition traits until now [6–11].

In recent years, the development of bioinformatics software and cost reduction of next generation sequencing (NGS) has opened a new era for genomics and molecular biology[12]. Compared to the traditional Sanger capillary electrophoresis sequencing method [13, 14], NGS technologies that is massively parallel DNA sequencing methods, provide higher throughput data with lower cost and make population-scale genome research possible[15–17]. Moreover, NGS can detect rare mutations, solve the disequilibrium between the rare causal mutations, genotype SNPs and distinguish structural variants [15, 18]. As for all kinds of variants, single nucleotide polymorphisms (SNPs) are the most widespread and wide-used in identification of genes for complex traits [19, 20]. Some whole genome resequencing studies in cattle have been reported on SNPs and copy number variations (CNVs) for genetic differences between the Black Angus and Holstein[21], Hanwoo-specific structural variations and selection signatures for meat quality and disease resistance traits in Hanwoo [22], haplotype under selection in USA Holstein [23] and evolutionary analysis in Japanese Kuchinoshima-Ushi [24]. In our previous studies, we detected some CNVs and insertions and deletions (indels) associated with milk protein and fat in Chinese Holstein [25, 26]. In the present study, we searched for differential SNVs between the Holstein bulls with extremely high and low estimated breeding values (EBVs) for milk protein percentage (PP) and fat percentage (FP) traits based on whole genome sequencing data, and identified candidate genes for milk compositions by integrating biological functions and the known QTL data.

## Materials and methods

### Sample selection and resequencing

Eight Holstein bulls were selected from the Beijing Dairy Cattle Center (http://www.bdcc.com.cn/) that consisted of four full-sib and/or half-sib families, and each family contain s two bulls who have extremely high and low EBVs for milk protein percentage (PP) and fat percentage (FP) with reliabilities of more than 0.85. The detailed information of the 8 bulls were described previously [25, 26].

The frozen semen samples were used for genomic DNA collection with the standard phenol/chloroform extraction method. 1% agarose gels and Nano Drop 2000 (Thermo Scientific Inc. Waltham, DE, USA) were performed for the DNA concentration and purity control. The purified DNAs were then used for library construction. Eight paired-end libraries (read length = 2×100 bp) with one library for each bull were constructed, and subsequently sequenced on Illumina Hiseq2000 instruments (Illumina Inc., San Diego, CA, USA).

### Read mapping and SNV calling

By using the Burrows–Wheeler Alignment tool (BWA ver. 0.6.2)[27], the sequenced reads were aligned to the bovine reference genome assembly UMD3.1.69 (ftp://ftp.ensembl.org/pub/release-69/fasta/bos_taurus/dna/) with the default parameters. NGS QC Toolkit with default parameters was applied to reduce mapping error rate [28]. By comparing 8 individual sequence to the bovine reference genome respectively, we called SNVs for each bull based on SAM tools (ver. 0.1.19)[29] with following criteria: base quality score ≥20; read depth <100 for each individual; and non-reference allele supporting reads >3. Based on this, 8 sets of SNV data for 8 bulls could be obtained.

### Functional annotation and SNV filtering

After SNV calling, the SNVs were annotated by ANNOVAR[30] using the RefSeq gene sets (14,912 genes; the gene sets is available from the UCSC download site http://hgdownload.cse.ucsc.edu/goldenPath/bosTau6/database/). The region that was close to a gene with less than 1kb was defined as upstream/downstream and that with more than 1kb was defined as intergenic region.

Afterwards, every single nucleotide which was polymorphic between the two bulls with high and low EBVs within each family was preserved. Then the SNVs with opposite fixed sites across four families were chosen and defined as ‘common differential SNVs’. Fixed sites, which were SNVs with opposite fixed alleles in the high and low group were used for identification of candidate genes.

### Functional enrichment analysis

After annotation, we selected the genes that included or were closed to the common differential SNVs with less than 5 kb. Then, we performed Gene Ontology (GO), Kyoto Encyclopedia of Genes and Genomes (KEGG) pathway and Medical Subject Headings (MeSH) enrichment for these genes. KOBAS tool(http://kobas.cbi.pku.edu.cn/) was used for GO and KEGG pathway enrichment and MeSH ORA was applied for MeSH enrichement[31–33].All packages used in MeSH analysis are available in the releases of BIOCONDUCTOR (http://bioconductor.org/). P value of <0.05 determined by Fisher’s exact test was set as the criteria for significance.

Apart from the genes that were referred in the significantly enriched pathways, we also remained the genes that were not significantly enriched but involved in the 8 well-known pathways related to protein, fat, and fatty acid metabolisms based on the KEGG pathway website (http://www.kegg.jp/), including mTOR, insulin, AMPK, PPAR, Jak-STAT, PI3K-Akt, MAPK, and TGF-β.

### Positions comparison with known QTL database

Afterwards, we obtained the genetic position of each gene based on its physical position and compared with the confidence intervals and the peak positions of the previously reported QTLs that have been shown to be associated with milk composition traits (http://www.animalgenome.org/cgi-bin/QTLdb). The genes that were close to the peaks of QTLs with less than 1 cM were remained.

## Results

### Read mapping and SNV detection

With Illumina HiSeq 2000, we sequenced the genomic DNA samples of the eight Holstein bulls with extremely high and low EBVs for milk protein percentage and milk fat percentage [25, 26]. As a result, a total of 2,303,781,449 raw reads were obtained. Of these, 2,055,337,835 reads (91.71%) were finally mapped to the reference genome (UMD 3.1.69) while the average proportion of uniquely mapped reads was 82.62% with an average depth of 8.1×, and the genome coverage was approximately 98% in each individual.

By using SAM tools with variant filtration process, 10,961,243 SNVs were identified in total after removing duplicates among 8 bulls at an average of 4,560,713 within each individual (Table 1).

**Table 1:** Summary of the sequencing result and SNV counts for 8 extreme Holstein bulls.

Sib-family	Sample	Raw reads	Mapped reads	Mapped reads (%)	Uniquely mapped reads (%)	Genome coverage (%)	Sequencing depth(X)	SNV
full-sib1	high 1	289,952,310	261,783,075	92.01	82.9	98.58	8	4,925,685
	low 1	286,870,238	252,201,294	90.24	80.88	98.55	8	4,636,009
full-sib2	high 2	292,878,886	257,840,281	91.28	82.97	98.59	8	5,075,588
	low 2	272,948,496	241,748,531	91.48	81.69	98.52	8	4,166,954
half-sib1	high 3	251,953,446	218,677,291	89.03	81.15	98.34	7	4,306,882
	low 3	337,815,303	314,651,325	96.73	83.75	98.4	10	5,403,284
half-sib2	high 4	288,003,254	253,311,627	91.01	82.45	98.61	8	4,572,191
	low 4	283,359,516	255,124,411	91.89	85.16	98.57	8	3,399,107
average		287,972,681	256,917,229	91.71	82.62	98.52	8.1	4,560,713

### SNV annotation and genomic distribution

**Fig 1:**
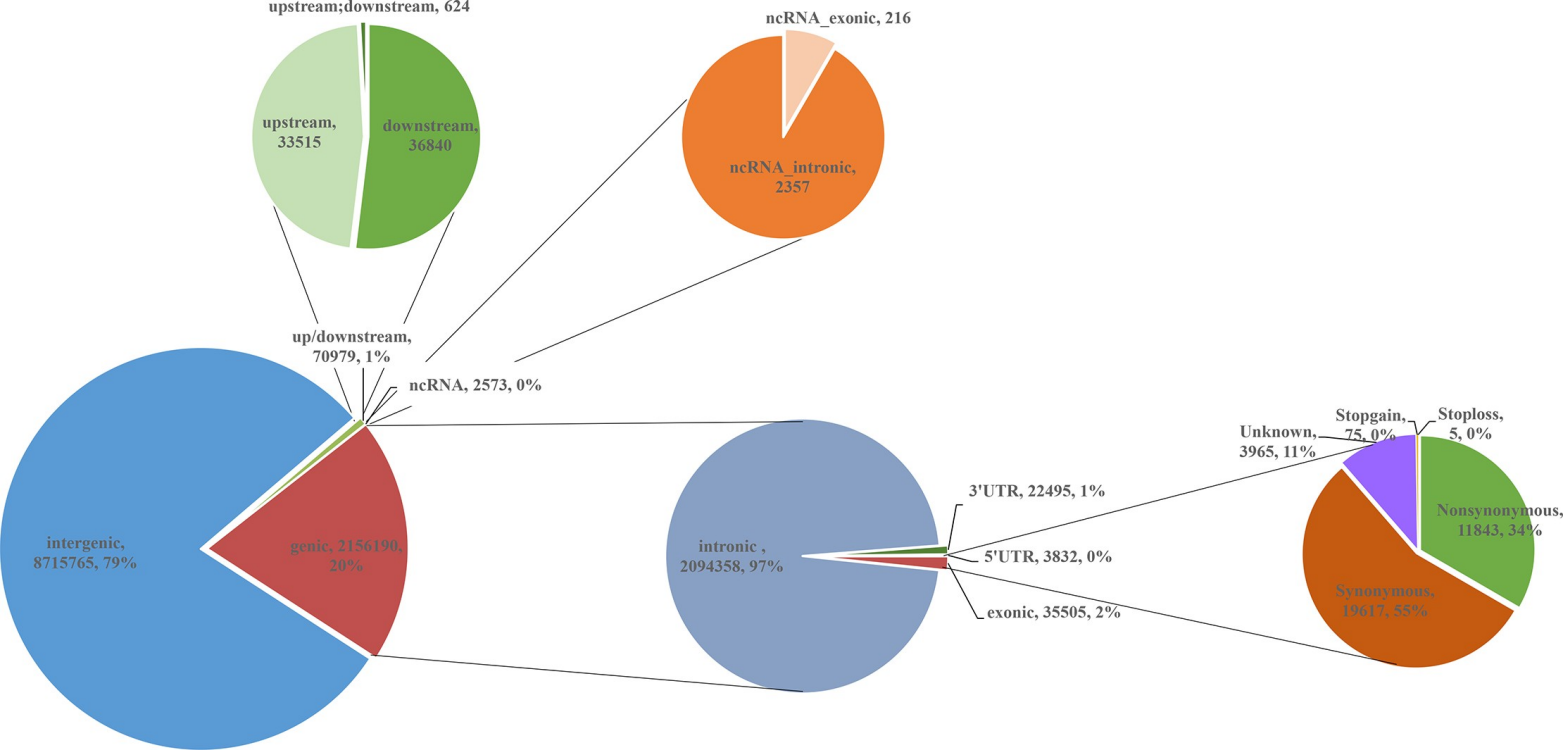
Distribution of the 10,945,507 annotated SNVs according to the functional category. After annotation of all SNVs among eight bulls, we found 8,715,765 intergenic SNVs (away from protein-coding genes more than 1 kb), 70,979 up/downstream SNVs, 2,573 ncRNA SNVs, 2,094,358 intronic SNVs, 26,327 untranslated regions (UTRs), 19,617 synonymous SNVs, 11,843 nonsynonymous substitutions, 75 stop gain SNVs, 5 stop loss SNVs and 3,965 unknown.

The 10,945,507 SNVs were annotated based on the bovine gene set in RefSeq database (including 14,912 genes) (S1 Table). Most of the SNVs (79.63%) were detected in intergenic region, and 2,156,190 (19.70%) SNVs were located within genes including introns (19.13%), exons (0.32%) and untranslated regions (UTR) (0.24%), and other region (0.68%) (ncRNA_exonic, ncRNA_intronic and up/downstream) (Table 2 and Fig 1). Of the total 35,505 exonic SNVs, 11,843 nonsynonymous nucleotide substitutions were included.

**Table 2:** Annotation of 10,945,507 SNVs across 8 bulls.

Category	Number of SNP	Percentage of SNP %^1^
intergenic	8,715,765	79.63
upstream^a^	33,515	0.31
downstream^b^	36,840	0.34
upstream and downstream^c^	624	0.01
3’ UTR	22,495	0.21
5’ UTR	3,832	0.04
ncRNA__exonic^d^	216	0.002
ncRNA__intronic^e^	2357	0.02
intronic	2,094,358	19.13
exonic		0.32
nonsynonymous	11,843	
synonymous	19,617	
stop gain	75	
stop loss	5	
unknown	3,965	

^1^Percentage was calculated based on total annotated SNVs.

^a^upstream from the nearest gene (<1kb).

^b^downstream away from the nearest gene (<1kb).

^c^variant located in both upstream and downstream regions for two different genes (<1kb).

^d^non-coding RNA expressed within exon of a gene

^e^non-coding RNA expressed within intron of a gene

### Identification of common differential SNVs

**Fig 2:**
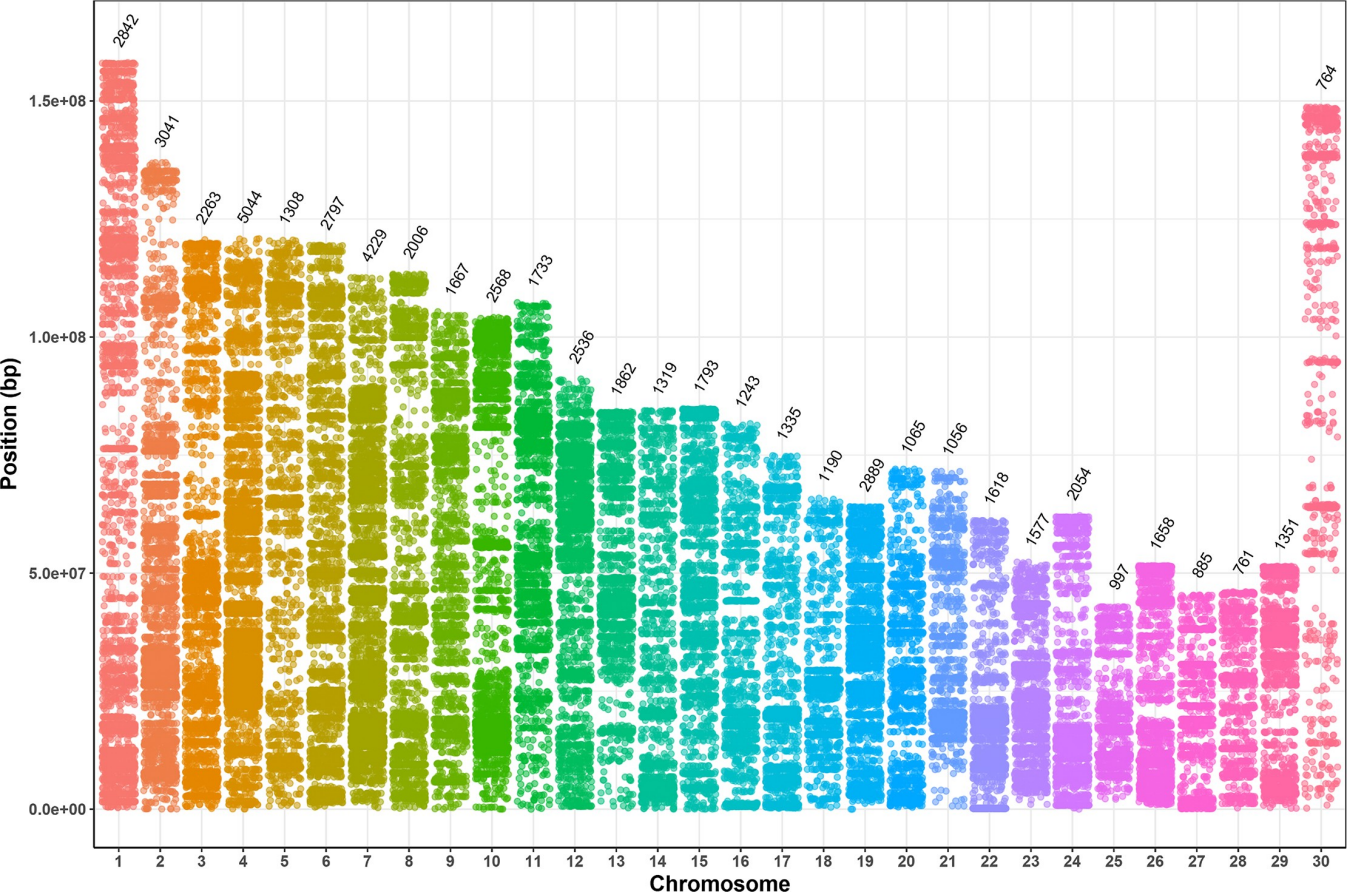
The number of common differential SNVs in each chromosome. Each point represents the location of a SNV on chromosome and the number above every chromosome represents the counts of SNV in this chromosome.

**Fig 3:**
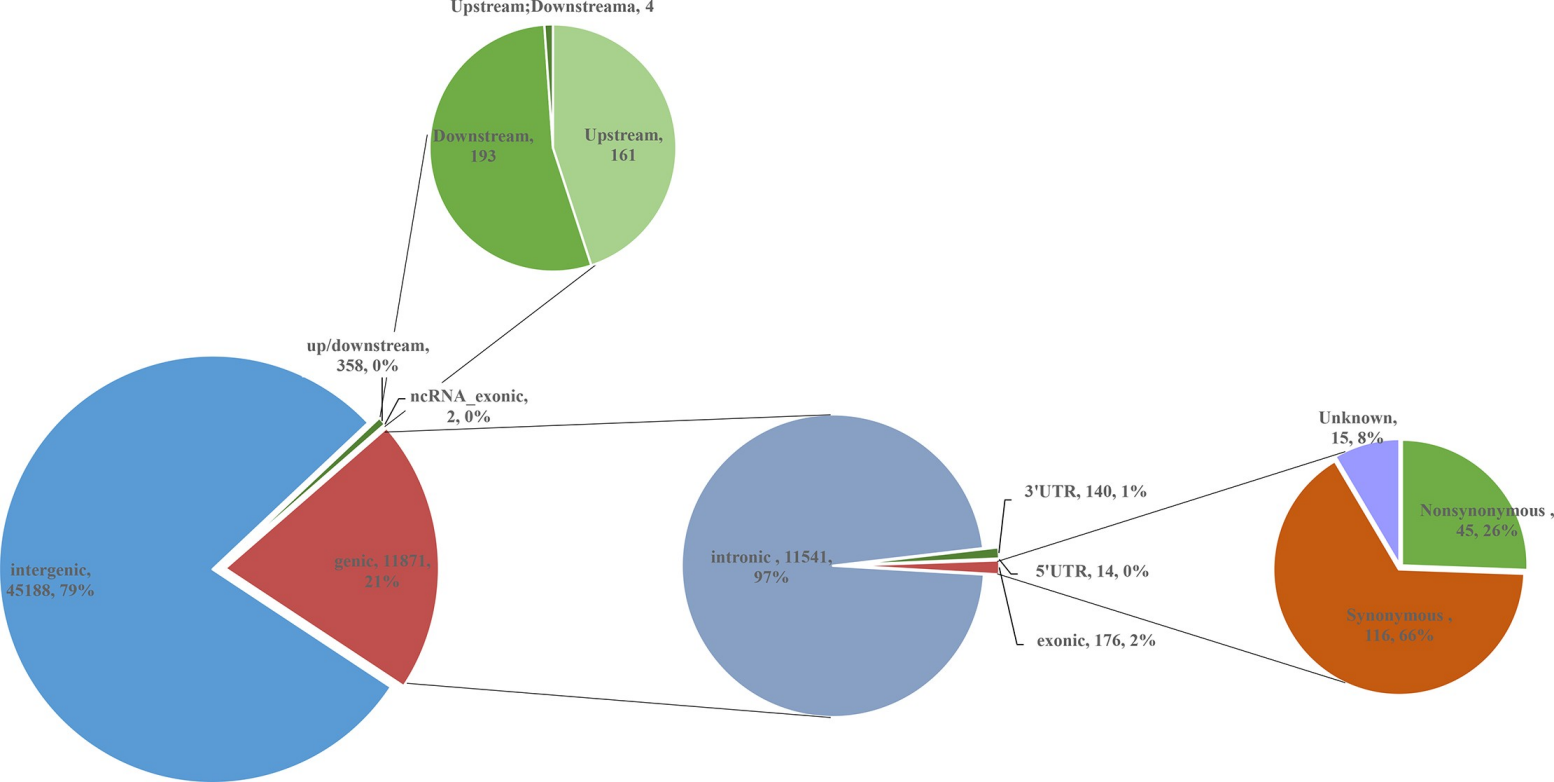
Distribution of the common differential SNVs in nine functional categories. After annotation, we found 45,188 intergenic SNVs (away from protein coding genes more than 1kb), 358 up/downstream SNVs, 2 ncRNA SNVs, 11,541 intronic SNVs, 154 untranslated regions (UTRs), 116 synonymous SNVs, 45 nonsynonymous substitutions and 15 unknown.

Out of the 10,945,507 annotated SNVs, 57,451 that were fixed sites between the bulls with extremely high and low EBVs across four families were chosen for the further analysis, which number across chromosomes ranged from 761 to 5,044 (Fig 2). As a result of annotation, 57,419 common differential SNVs were successfully classified into 9 functional categories: the majority was found in intergenic and intronic regions (78.70% and 20.10%, respectively), whereas fewer SNVs were located in exon (0.31%), exonic ncRNA (0.003%), UTR (0.26%) and up/downstream (0.63%) (Table 3 and Fig 3).

**Table 3:** Annotation of 57,419 common differential SNVs across 8 bulls.

Category	Number of SNVs	Percentage of SNVs %^1^
intergenic	45,188	78.70
upstream^a^	161	0.28
downstream^b^	193	0.34
upstream;downstream^c^	4	0.01
3’UTR	140	0.24
5’UTR	14	0.02
ncRNA_exonic^d^	2	0.003
intronic	11,541	20.10
exonic		0.31
nonsynonymous	45	
synonymous	116	
unknown	15	

^1^Percentage was calculated based on annotated 57,419 common differential SNVs.

^a^upstream from the nearest gene (<1kb).

^b^downstream away from the nearest gene (<1kb).

^c^variant located in both upstream and downstream regions for two different genes (<1kb).

^d^non-coding RNA expressed within exon of a gene

**Fig 4:**
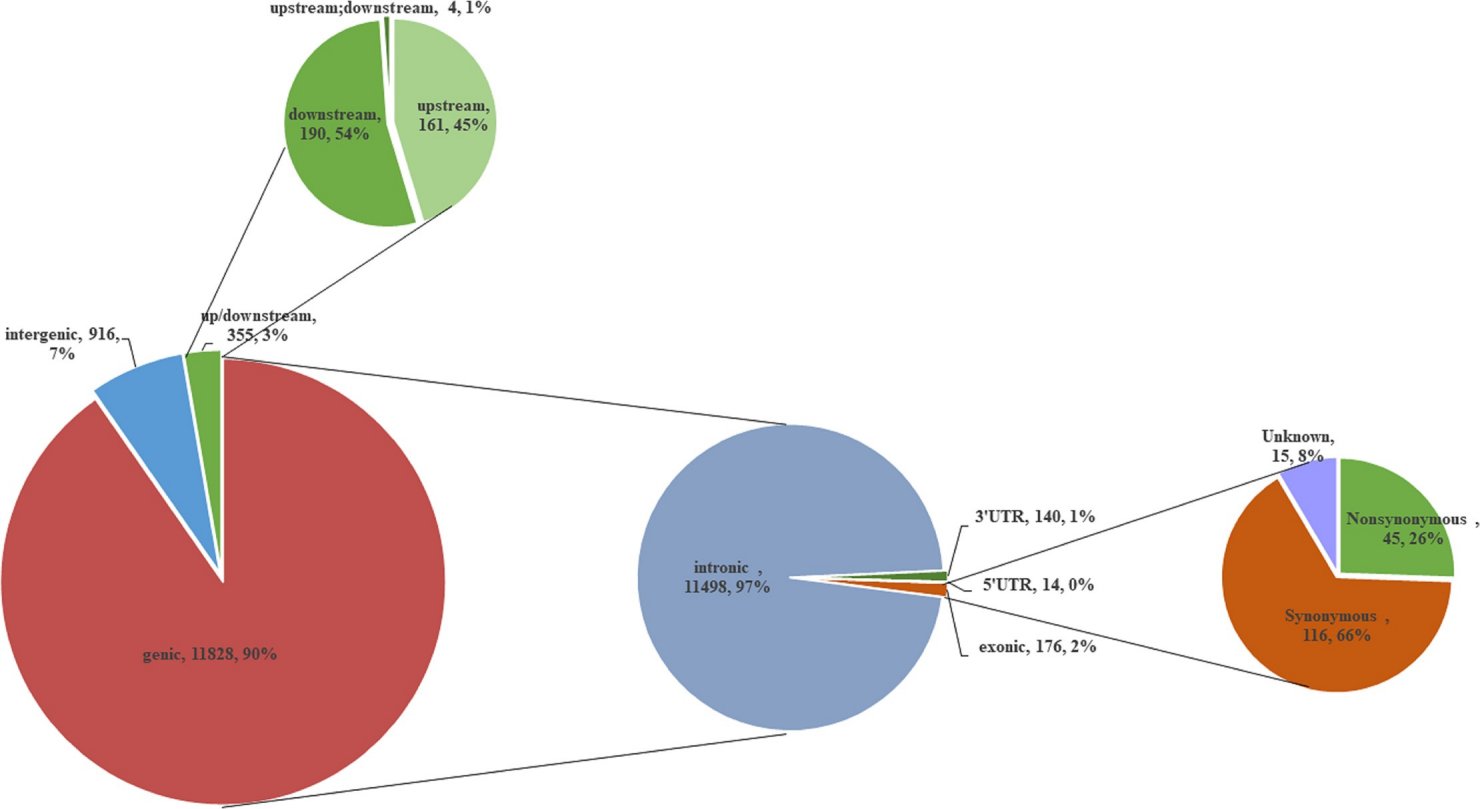
Distribution of the 13,099 SNVs in eight functional categories. A total of 13,099 SNVs was included or nearby the protein-coding genes with less than 5 kb. Of these, 916 SNVs were located in intergenic region (away from protein coding genes more than 1kb), 355 in up/downstream, 11,498 in intron, 154 untranslated regions (UTRs), 176 in exon (116 synonymous SNVs, 45 nonsynonymous substitutions and 15 unknown).

Subsequently, we further identified 2,657 protein-coding genes that included or were nearby the common differential SNVs with less than 5 kb. Of these, 13,099 SNVs were remained, including 11,498 (87.78%) in intron, 176 (1.34%) in exon, 154 (1.18%) in UTR, and 355 (2.71%) in upstream/downstream while only 6.99% were detected in intergenic region (Table 4 and Fig 4).

**Table 4:** Annotation of 13,099 common differential SNVs that were included or nearby 2,657 protein-coding genes.

Category	Number of SNVs	Percentage of SNVs %^1^
intregenic	916	6.99
upstream^a^	161	1.23
downstream^b^	190	1.45
upstream;downstream^c^	4	0.03
3’UTR	140	0.11
5’UTR	14	1.07
intronic	11,498	87.78
exonic		1.34
nonsynonymous	45	
synonymous	116	
unknown	15	

^1^Percentage was calculated based on 13,099 SNVs.

^a^upstream from the nearest gene (<1kb).

^b^downstream away from the nearest gene (<1kb).

^c^variant located in both upstream and downstream regions for two different genes (<1kb).

### Genes Ontology and pathway analyses

To further identify candidate genes for milk protein and fat traits, we performed functional analysis on the above-mentioned 2,657 genes with KOBAS online tool and MeSH ORA. A total of 6,819 GO terms and 286 KEGG pathways were observed, among them with 1,011 terms and 73 pathways were significantly enriched (P<0.05; S2 Table). 23 significant MeSH terms were detected by MeSH ORA(P<0.05; S3 Table). Of these, 29 genes was enriched in Mesh term of Amino Acids (MeSH:D000596) in the Chemicals and Drugs category which was associated with protein synthesis and metabolism Thereby, we identified 1,354 genes that were involved in 133 significant GO terms, pathways and Mesh terms relevant to protein, lipid, and fatty acid synthesis and metabolism such as protein metabolic, cellular protein modification, lipid modification, phospholipid metabolic, glycerophospholipid metabolic, sphingolipid metabolism, glycerolipid metabolic, fat cell differentiation, insulin resistance, insulin secretion and MAPK signaling pathways.

Besides the genes which were significantly enriched in pathways associated with protein and fat synthesis and metabolism, we selected 23 additional genes that were not significantly enriched but participated in six well-known pathways such as mTOR, AMPK, Jak-STAT, PI3K-Akt, PPAR and TGF-β.Thus, 1,377 candidate genes were obtained for milk protein and fat traits.

### Position comparison with known QTLs and identification of promising candidates associated with milk protein and fat traits

We further compared the physical positions of the 1,377 candidate genes with the previously reported QTLs for milk fat and protein in dairy cattle (http://www.animalgenome.org/cgi-bin/QTLdb). Consequently, 94 genes were found to be adjacent to the peak positions of QTLs with less than 1.0 cM. Of these, 17 genes with 21 common differential SNVs in exon, UTR, upstream and downsteam were identified as promising candidates affecting milk protein and fat traits. They included UDP-N-acetylglucosaminyltransferase subunit (*ALG14*), ATPase secretory pathway Ca2+ transporting 1 (*ATP2C1*), phosphatidylcholine-specific (*PLD1*), glycosylated lysosomal membrane protein (*C3H1orf85*), sorting nexin 7 (*SNX7*), methylenetetrahydrofolate dehydrogenase (NADP+ dependent) 2 like (*MTHFD2L*), cyclin dependent kinase inhibitor 2D (*CDKN2D*), alpha3 (V) collagen chain (*COL5A3*), ferredoxin 1-like(*FDX1L*), NIMA-interacting 1(*PIN1*), phosphoinositide 5-phosphatase (*FIG4*), exocyst complex component 7 (*EXOC7*), LIM and SH3 protein 1 (*LASP1*), phosphatidylglycerophosphate synthase 1 (*PGS1*), primary amine oxidase, liver isozyme (*SAO*), glycosylphosphatidylinositol specific phospholipase D1 (*GPLD1*) and OGA O-GlcNAcase *(MGEA5*). The alleles of the common differential SNV in high and low groups and the adjacent QTLs of 17 candidate genes were shown in Tables 5 and 6, respectively.

**Table 5:** The information about the SNV allele in high and low groups of 17 candidate genes.

Gene	Gene start^1^	Gene end	Chromosome	Location of SNV	Physical position of SNV	SNV allele in high group	SNV allele in low group
ALG14	48600669	48699217	1	UTR5	48600677	T	C
ATP2C1	140368052	140522627	1	exonic	140375966	A	G
			1	exonic	140388073	A	G
PLD1	96517508	96676253	1	exonic	96594836	C	T
C3H1orf85	14558546	14561400	3	upstream	14558465	T	C
SNX7	44657521	44775833	3	downstream	44656702	A	G
MTHFD2L	90842884	90986715	6	downstream	90987596	A	G
CDKN2D	16298106	16300588	7	UTR3	16298320	G	T
COL5A3	15768627	15813561	7	exonic	15769886	G	A
FDX1L	16068661	16073072	7	UTR3	16068671	C	G
PIN1	15532997	15545250	7	UTR3	15544947	C	T
			7	UTR3	15544948	C	T
FIG4	40873044	41046003	9	exonic	40957415	G	A
EXOC7	56190845	56208380	19	exonic	56206567	G	A
LASP1	40090994	40131374	19	UTR3	40129782	C	G
			19	downstream	40131920	A	G
PGS1	54404339	54441590	19	downstream	54404267	T	C
SAO	43555807	43559785	19	downstream	43560755	A	G
			19	downstream	43560756	A	C
GPLD1	32984455	33036038	23	upstream	32984009	C	T
MGEA5	22390769	22417775	26	upstream	22418458	A	G

^1^genomic coordinates of genes were based on Bos_taurus_UMD_3.1

**Table 6:** The detailed information of 17 candidate genes and the related QTLs.

Gene	Position(bp)	Position(cM)^1^	Previously reported QTL			
			Distance to QTL peak (cM)	CI and peak location(cM)	Trait	Reference
*ALG14*	Chr1:48600669–48699217	Chr3:48.4	0	48.37–48.37(peak:48.37)	PY	Marete et al., Frontiers in genetics, 2018[34]
*ATP2C1*	Chr1:140368052–140522627	Chr1:134.9	0.3	134.24–135.04 (peak:134.64)	PP	Russo et al., Animal genetics, 2012[35]
*PLD1*	Chr1:96517508–96676253	Chr1:93.7	0.2	77.7–122.3(peak:93.5)	PY	Nadesalingam et al., Mammalian genome,2001[36]
*C3H1orf85*	Chr3:14558546–14561400	Chr3:24.3	0.7	22.6–27.4(peak:25)	FY,PP	Ashwell et al., J Dairy Sci,2004[37]
*SNX7*	Chr3:44657521–44775833	Chr3:46.7	0.8	45.93–45.93(peak:45.95)	FY	Cole JB et al., BMC Genomics, 2011[38]
				45.93–45.93(peak:45.95)	PY	Cole JB et al., BMC Genomics, 2011[38]
*MTHFD2L*	Chr6:90842884–90986715	Chr6:100	0.9	100.09–100.09 (peak:100.9)	PP	Olsen HG et al., Genetics, selection, evolution : GSE, 2016[39]
			0.4	100.28–100.49 (peak:100.38)	PP	Zhou Y et al., BMC genomics, 2018[40]
*CDKN2D*	Chr7:16298106–16300588	Chr7:18.4	0.6	15.2–38.5(peak:17.8,15.9)	PP	Ron et al., Journal of dairy science, 2004[41]
*COL5A3*	Chr7:15768627–15813561	Chr7:17.9	0.1	15.2–38.5(peak:17.8,15.9)	PP	Ron et al., Journal of dairy science, 2004[41]
*FDX1L*	Chr7:16068661–16073072	Chr7:18.2	0.4	15.2–38.5(peak:15.9,17.8)	PP	Ron et al., Journal of dairy science,2004[41]
*PIN1*	Chr7:15532997–15545250	Chr7:17.5	0.3	15.2–38.5(peak:17.8,15.9)	PP	Ron et al., Journal of dairy science,2004[41]
*FIG4*	Chr9:40873044–41046003	Chr9:45	0.6	42.5-50(peak:44.4,49.1)	FY,PY	Schnabel et al., Animal Genetics, 2005[42]
*EXOC7*	Chr19:56190845–56208380	Chr19:96.1	0.9	82.8–101.4(peak:95.2)	PY	Boichard et al., Genetics,selection,evolution:GSE, 2003[43]
*LASP1*	Chr19:40090994–40131374	Chr19:51.3	0.7	2.4-91(peak:60.4)	FP	Bennewitz et al., Genetics, 2004[44]
*PGS1*	Chr19:54404339–54441590	Chr19:93.1	0.7	60.7–106.2(PEAK:92.4)	FY	Boichard et al., Genetics,selection,evolution:GSE, 2003[43]
*SAO*	Chr19:43555807–43559785	Chr19:74.5	0.5	70.24–77.386 (peak:75.0)	FP	Viitala SM et al., J Dairy Sci, 2003[45]
*GPLD1*	Chr23:32984455–33036038	Chr23:44.2	0.6	42.4–58.2(peak:43.6)	FY	Plante et al.,J Dairy Sci,2001[46]
*MGEA5*	Chr26:22390769–22417775	Chr26:32.5	0.8	31.72–31.72(peak:31.72)	PP	Cole JB, et al, BMC Genomics, 2011[38]

^1^The linkage position was estimated relative to UMD3.1 and based on the QTL mapper v.2.019 at www.animalgenome.org/cgi-bin/QTLdb/.

PP: protein percentage; PY: protein yield; FP: fat percentage; FY: fat yield.

## Discussion

In this study, based on the whole genome resequencing data of 8 proven Holstein bulls from the four half-sib or full-sib families with extremely high and low EBVs for milk protein and fat percentages, we obtained 57,419 common differential SNVs between high and low groups, and further identified 17 promising candidate genes for milk composition traits by integrating the positions of SNVs in gene regions, the known QTLs and the biological functions of genes.

Since the first report of SNV detection by the whole genome resequencing in cattle, a number of SNVs have been detected in different cattle breeds. In this study, a total of 10,961,243 SNVs were identified in 8 Holstein bulls (average 4,560,713 for each), which was much more than those in Holstein bulls reported by Paul et al. (SNP = 3,755,663) [21], but fewer than other Holstein bulls studies(SNP = 12,434,860 and 26.7 million, respectively)[23, 47]. This was probably due to the different sequencing depth and coverage.

### Candidate genes

The 17 identified promising candidate genes that contained one or two common differential SNVs were specifically illustrated follow.

#### Candidate genes with SNV in exon

SNV in exon of a gene, especially nonsynonymous variants, potentially had a bigger influence on gene function. *COL5A3* with a nonsynonymous SNV in exon 2 encodes collagen type V alpha 3 belong to a superfamily of proteins. *COL5A3* takes part in protein digestion, absorption and PI3K-Akt signaling pathway. Previous study found that obese black women exhibited higher expression of *COL5A1* (collagen Valpha1), and *COL6A1* (collagen VIalpha1) than obese white women in gluteal [48]. In our previous RNA sequencing study among 3 milking period Holstein cattle, Collagen VI was found involved in regulating fat metabolism[49]. *COL5A3* is an important element of the microenvironment of certain highly specialized cell types in white adipose tissue and have profound effects on function of such cells [50]. Actually, nonsynonymous and synonymous coding SNPs show similar likelihood and effect size of traits [51–54]. *ATP2C1* with 2 synonymous SNVs encodes a protein belongs to the family of P-type cation transport ATPases which catalyzes the hydrolysis of ATP coupled with the transport of calcium ions. *ATP2C1* activity is associated with the sphingomyelin content of the trans-Golgi network membrane and it regulates proteases within the trans-Golgi network that require for virus glycoprotein maturation [55, 56]. The study of rat demonstrated that *ATP2C1* played a role in the control of beta-cell Ca (2+) homeostasis and insulin secretion [57]. In addition, Golgi Ca2+/H+ antiporter as a contributor to mammary Golgi calcium transport needs was related to the role of *ATP2C1* and *ATP2C2 [58]*. *PLD1* encodes a phosphatidylcholine-specific phospholipase which catalyzes the hydrolysis of phosphatidylcholine in order to yield phosphatidic acid and choline. The deficiency of *PLD1* or *PLD2* activity promotes elevated free fatty acids (FFA) levels and are insulin as well as glucose intolerant[59]. Besides, *PLD1* regulates COPII vesicle transport from the endoplasmic reticulum (ER) to the Golgi apparatus by regulating Sec13/31 recruitment from the cytosol to the ER membrane during COPII vesicle formation [60]. *EXOC7* encodes a protein which is a component of the exocyst complex that plays a critical role in vesicular trafficking and the secretory pathway by targeting post-Golgi vesicles to the plasma membrane. *EXOC7* is a direct substrate of the extracellular signal-regulated kinases 1/2, their phosphorylation enhances the binding of *EXOC7* to other exocyst components and promotes the assembly of the exocyst complex [61, 62]. PIPKIgamma and phosphatidyl inositol phosphate pools at nascent E-cadherin contacts cue *EXOC7* targeting and orient the tethering of exocyst-associated E-cadherin [62]. The protein encoded by *FIG4* belongs to the SAC domain-containing protein gene family. *FIG4* binds to hepatitis C virus and modulates particle formation in a cholesteryl ester-related manner [63].

#### Candidate genes with SNV in regulatory regions

SNV in regulatory regions probably regulates the translation processes of a gene. *ALG14* with a SNV in 5’UTR is a member of the glycosyltransferase 1 family. The protein encoded by *ALG14* and *ALG13* are thought to be subunits of UDP-GlcNAc transferase, which catalyzes the first two committed steps in endoplasmic reticulum N-linked glycosylation. *ALG14* coordinate recruitment of catalytic *ALG7* and *ALG13* to the endoplasmic reticulum membrane for initiating lipid-linked oligosaccharide biosynthesis at the N- and C-termini and interacted formation of the active UDP-N-acetylglucosamine transferase complex at the C terminus mediates[64, 65]. *CDKN2D*, *LASP1* and *PIN1* respectively contained 1, 1 and 2 SNVs in 3’UTR. *CDKN2D* encoded a protein which is a member of the INK4 family of cyclin-dependent kinase inhibitors that form a stable complex with *CDK4* or *CDK6*, and prevent the activation of the CDK kinases, thus function as a cell growth regulator that controls cell cycle G1 progression. *FDX1L* encodes a member of the ferredoxin family. The mutation of genes that encoded proteins involved in either the lipoic acid (*LIPT1* and *LIPT2*) or mitochondrial ISC biogenesis (*FDX1L*, *ISCA2*, *IBA57*, *NFU1*, *BOLA3*) pathway leaded a heterogeneous group of diseases with a wide variety of clinical symptoms and combined enzymatic defects [66]. The protein encoded by *LASP1* is a subfamily of LIM proteins and also a member of the nebulin family of actin-binding proteins. *LASP1* activates the PI3K/AKT signaling pathway which is well-known pathways for protein and fat synthesis and metabolism [67]. *LASP1* was significantly upregulated in breast cancer tissues and cell lines and identified as a target gene of miR-133a [68]. Comparing gene expression profiles of lactating bovine mammary tissue against nonlactating tissue on the BMAM microarray, *LASP1* exhibited differential expression [69]. *PIN1* encodes one of the PPIases, which specifically binds to phosphorylated ser/thr-pro motifs to catalytically regulate the post-phosphorylation conformation of its substrates and involved in the regulation of cell growth. Besides, *PIN1* can enhance adipocyte differentiation by regulating the function of PPAR gamma [70]. Another study suggested that *PIN1* expression in pancreatic beta-cells was obviously changed in obese knockout mice from diet high in fat or sucrose [71].

#### Candidate genes with SNV in upstream and downstream

Transcription factors interact with specific nucleotide sequences known as transcription factor binding site and these interactions are implicated in regulation of the gene expression. The upstream and downstream regions of genes contain variety of elements/binding sites, which apparently infer on a particular gene the inducibility. *C3H1orf85*, *GPLD1*, *MTHFD2L*, *LASP1*, *MGEA5*, *PGS1*, *SAO*, *SNX7* contained at least one SNV located in upstream and downstream (<1000 bp) of these gene. *C3H1orf85* encodes glycosylated lysosomal membrane protein and was also known as *GLMP*. Data indicated that increased flux of glucose, increased de novo lipogenesis and lipid accumulation were detected in lysosomal protein NCU-G1 (*GLMP*) gt/gt primary hepatocytes[72]. Compared with the wild-type myotubes, myotubes from *GLMP* (gt/gt) mice metabolized glucose faster and had a larger pool of intracellular glycogen, while oleic acid uptake, storage and oxidation were significantly reduced [73]. The nuclear proteins by O-linked N-acetylglucosamine (*MGEA5*) addition and removal on serine and threonine residues is catalyzed by OGT (MIM 300255), which adds *O-GlcNAc*, and *MGEA5*, a glycosidase that removes O-GlcNAc modifications[74]. *PGS1* encodes a phosphatidylglycero-phosphate synthase. In cancer cachexia, TNFalpha induces a higher energy wasting in liver mitochondria by increasing cardiolipin content via upregulation of phosphatidylglycerophosphate synthase (*PGPS*) expression [75]. *PGS1* gene in Saccharomyces cerevisiae played a vital role in cells impaired in the mitochondrial DNA, is localized in the mitochondria and expressed in response to inositol and choline[76]. The protein encoded by *SAO* is part of the anion exchanger (AE) family and is expressed in the erythrocyte plasma membrane. *MTHFD2L* encodes a mitochondrial methylenetetrahydrofolate dehydrogenase isozyme expressed in adult tissues. *SNX7* encodes a member of the sorting nexin family that contain a phox (PX) domain, which is a phosphoinositide binding domain, and are involved in intracellular trafficking. In zebrafish, *SNX7* is a liver-enriched anti-apoptotic protein and indispensible for the liver development[77]. The protein encoded by *GPLD1* is a GPI degrading enzyme. *GPLD1* hydrolyzes the inositol phosphate linkage in proteins anchored by phosphatidylinositol glycans, thereby releasing the attached protein from the plasma membrane. AMPK suppresses *PLD* activity, and *PLD* suppresses AMPK via mTOR [78]. Additionally, *GPLD1* influences triglyceride-rich lipoprotein metabolism [79]. Overexpressing *GPLD1* in an insulinoma cell line enhanced glucose-stimulated insulin secretion [79].

The 17 candidate genes and 21 SNVs identified in this study still need further in vivo and in vitro experiments to validate their biological function and to explore molecular mechanisms for formation of milk protein and fat traits.

The interpretation of the findings from the present study still has limitations. When performed function enrichment for genes that included or were closed to the common differential SNVs with less than 5 kb, non-coding RNAs and genes could be disregarded because the current software and tools can only annotate limited protein-coding genes. Therefore, the omission of genes that haven not been studied yet is a general problem in present function study.

## Conclusions

In this study, by resequencing the whole genome of eight proven Holstein bulls with extremely high and low EBVs of milk protein percentage and fat percentage, we successfully identified 10,961,243 SNVs and detected 57,451 common differential SNVs with opposite fixed sites between high and low groups. Subsequently, 2,657 genes that included or were nearby the common differential SNVs were obtained. Further, through integrating GO, KEGG pathways and Mesh enrichment results, the known QTLs for milk composition and common differential SNVs located in exon and flanking regions, we identified 17 promising candidate genes for milk protein and fat, including *ALG14*, *ATP2C1*, *PLD1*, *C3H1orf85*, *SNX7*, *MTHFD2L*, *CDKN2D*, *COL5A3*, *FDX1L*, *PIN1*, *FIG4*, *EXOC7*, *LASP1*, *PGS1*, *SAO*, *GPLD1* and *MGEA5*. And the 17 genes identified in this study will provide a useful resource for future genomic selection (GS) in dairy cattle.

### Ethics statement

All protocols for semen samples of China Holstein bulls were reviewed and approved by the Institutional Animal Care and Use Committee (IACUC) at China Agricultural University. Semen samples were collected specifically for this study following standard procedures with the full agreement of the Beijing Dairy Cattle Center who owned the animals.

## Supporting information

S1 Table Functional annotation of each identified SNVs. (ZIP)

S2 Table GO and KEGG pathways enrichment results for 2,657 genes by KOBAS tool. (XLSX)

S3 Table Mesh enrichment results for 2,657 genes by MeSH ORA. (XLSX)
