# Correction: SNV discovery and functional candidate gene identification for milk composition based on whole genome resequencing of Holstein bulls with extremely high and low breeding values

**DOI:** 10.1371/journal.pone.0225747

**Published:** 2019-11-20

**Authors:** Shan Lin, Hongyan Zhang, Yali Hou, Lin Liu, Wenhui Li, Jianping Jiang, Bo Han, Shengli Zhang, Dongxiao Sun

The Data Availability statement for this paper is incorrect. The correct statement is: The re-sequencing data for the eight Holstein bulls used in this paper have been deposited in NCBI and can be accessed in the Sequence Read Archive (SRA) repository under the accession numbers: SRX2254497, SRX2254776, SRX2254777, SRX2254778, SRX2254779, SRX2254780, SRX2254813 and SRX2254814.
